# The Making of a Radical: The Role of Peer Harassment in Youth Political Radicalism

**DOI:** 10.1177/01461672211070420

**Published:** 2022-01-29

**Authors:** Marta Miklikowska, Katarzyna Jasko, Ales Kudrnac

**Affiliations:** 1 Umeå University, Sweden; 2 Jagiellonian University, Poland

**Keywords:** political radicalism, youth radicalization, peer harassment, adolescents

## Abstract

Although political radicalism is one of the major societal threats, we have limited understanding of how it is formed. While there are reasons to expect that harassment experienced in adolescence increase the propensity for radicalism, this relationship has not yet been investigated. This five-wave study of Swedish adolescents (*N* = 892) examined the role of peer harassment in radical political behavior. The results revealed that within-person fluctuations in harassment were positively related to fluctuations in radicalism. Individual-level (but not class-level) harassment also predicted differences between adolescents: youth who experienced more harassment had higher levels of and a more pronounced decrease in radicalism. In addition, adolescents who had more supportive teachers or parents were less affected by harassment than youth with less-supportive adults. The findings suggest that personal experiences of harassment increase the risk of radicalism but supportive relationships can mitigate their negative consequences.

What do you get when you cross a (mentally-ill) loner with a society that abandons him and treats him like a trash?

—*Joker* (2019)

Political socialization and developmental theories suggest that adolescence is crucial for the formation of political behaviors and social identity (Erikson, 1968; Krosnick & Duane, 1989), including radical beliefs and actions. Moreover, this process seems to be more dynamic during that period than at other life stages. For example, whereas adults’ approval for violent political action was found to be stable (Rink & Sharma, 2016), youth approval fluctuated over time (Dahl, 2019; Weber, 2018). This suggests that attitudes toward radicalism undergo the most changes during adolescence and, thus, might be the most susceptible to social influence. Furthermore, studies found that adolescents expressed more approval of radical political action compared to adults (Watts, 1999). Given the low average age of extremists (e.g., 19 in Sweden; Rostami et al., 2016), the European Commission emphasized the need to focus prevention efforts on adolescents (European Commission, 2016).

Although adolescence seems to be an important period for radicalization, research on youth radicalization is scarce and limited to cross-sectional measures of attitudes toward political violence (e.g., Doosje et al., 2012, 2013) or behavioral intentions (Šerek, Machackova, & Macek, 2018) rather than actual radical political behaviors tracked over time. Moreover, it is unclear what factors motivate or help to prevent youth political radicalism. The present research addresses these gaps.

Drawing on the significance quest theory (Kruglanski et al., 2013, 2018), which identifies radical political behavior as a means to restore a diminished sense of self-worth, we examine the role of harassment in youth radicalism. Specifically, we test the long-term effects of peer harassment on radical political behavior in a 5-year panel of Swedish adolescents. Moreover, we examine whether having supportive relationships with teachers or parents helps to mitigate the negative consequences of harassment. Although experiences that boost social belonging have been suggested to counteract significance loss, and thus political radicalism, we could not identify any studies that tested this possibility in the context of real-life political radicalism.

## The Motivational Underpinnings of Political Radicalism

Radical political behavior is defined as an action that deviates from societal norms that describe what political goals and means of their attainment are appropriate (Kruglanski et al., 2018). As the strength of social norms is different for different behaviors, radicalism can also be conceptualized as a continuum. On one end of this continuum are mainstream political behaviors, which are approved by most people in society (e.g., signing a petition). The other end is represented by violent political actions, which are almost universally condemned (e.g., terrorism). Between these two endpoints, nonnormative actions of varying levels of aggression (e.g., destruction of property) can be found.

Given that deviation from social norms is effortful, motivational accounts of political radicalism have been proposed. These accounts suggest that radicalism is motivated by frustrated psychological needs, and the strength of the frustrated needs determines the extent of sacrifice one is willing to make for a political cause. In that regard, various psychological needs have been investigated in relation to political radicalism. For example, research on *belonging needs* demonstrated that when people do not feel accepted aggressive behavior is more likely, including politically motivated aggression. In line with that, a study on solo terrorist found that they were disproportionately likely to be socially isolated (Gill et al., 2014; Gruenewald et al., 2013). Another study showed that individuals who engaged in violence for political aims experienced more frustrations in their social relationships (Jasko et al., 2020). Similarly, research that focused on *epistemic needs* showed that when people crave certainty they are more likely to support extreme means and engage in extreme actions (Hogg et al., 2007, 2013; Webber et al., 2018). Furthermore, studies on *social identity needs* show that when people feel their ingroup is not treated fairly, support for political violence is more likely (Piazza, 2011; Victoroff et al., 2012).

Significance quest theory (Jasko et al., 2020; Kruglanski et al., 2017) builds upon these findings by proposing a common mechanism that underlies these effects. According to this account, other people’s harmful actions such as social rejection, discrimination, and harassment influence the extent to which one feels respected, recognized, and valued. Those experiences feed into one’s need to feel significant by creating a discrepancy between a desired positive view of oneself and a negative self-evaluation that is a result of those hurtful social experiences. This discrepancy becomes a motivating force for restoring a sense of self-worth. Radical political engagement offers an antidote to significant loss by making individuals feel noticed and engaged in a meaningful cause.

Empirical support for significance quest theory has been obtained in diverse settings (Dugas et al., 2016; Jasko et al., 2019; Lyons-Padilla et al., 2015; Schumpe et al., 2020; Webber et al., 2018). However, although past research on radicalism used diverse methodologies such as qualitative and cross-sectional data, there is no study tracking radicalism over an extended period of time using behavioral measures. Hence, we know little about the temporal dynamics of political radicalism. Moreover, while there is an abundance of research among adults, research on adolescents is scarce. At the same time, there are several arguments for why adolescence is important for the process of radicalization, given the role of frustrated psychological needs in this process.

## Psychological Needs and Political Radicalism in Adolescence

Adolescence is a sensitive period for radicalization. First, adolescents are the most vulnerable to frustrations of their belonging needs with peers given that the salience of peers as a reference group peaks in adolescence (Steinberg & Monahan, 2007) and that youth experience more peer harassment than children or adults (Eslea & Rees, 2001). As adolescents grapple with complex peer relations, they are still developing cognitive control over their behavior (Casey & Caudle, 2013), increasing their risk-taking and struggling to regulate emotional reactions to social rejection (Silvers et al., 2012). Thus, they are particularly prone to experience heightened negative effects of social rejection such as significance loss. Second, adolescence is a formative period for cultivating a sense of purpose and the development of social identity (Erikson, 1968; Loevinger, 1976). Adolescents search for a sense of self through an intense exploration of personal values, goals, and “possible selves” and they begin to dedicate themselves to grand systems of beliefs and purpose beyond self-advancement (Damon et al., 2003). Increased risk-taking is often interpreted as a normative pattern toward autonomy and identity development (Crone et al., 2016). In response to frustration of significance needs due to social rejection and identity crisis, youth are at heightened risk of turning to different, radical, worldviews, and lifestyles (Arnett, 2000; Kroger, 2004).

While these results suggest that adolescence is a sensitive period for radicalization, no studies examined changes in youth political radicalism over time. However, research on externalizing behaviors such as aggression and delinquency shows that such behaviors peak in early to mid-adolescence and decline after the age of 15 or 16 (Bongers et al., 2004; Lacourse et al., 2003; Moffitt, 2018), which suggests that radical activism should decrease in late adolescence as well. This decrease would be in line with older adolescents gaining more cognitive control over their behavior and emotional regulation (Casey & Caudle, 2013; Silvers et al., 2012) as well as developing a sense of identity (Crone et al., 2016). This would also be in line with decreasing association with delinquent peer groups (Elliott & Menard, 1996; Warr, 1993), following a decrease in general peer influence after the age of 14 (Steinberg & Monahan, 2007).

Similarly, few studies examined youth political radicalism in relation to their motives. Those that did have predominantly focused on frustrations of *belonging needs* related to collective identity. They found that youth who perceived their ethnic group to be discriminated against were more likely to support radical right-wing attitudes (Doosje et al., 2012), justify extremism (Kamans et al., 2009), and engage in political vandalism (Pauwels & De Waele, 2014). In contrast to research on collective frustrations, we found only one study that looked at the role of individual harassment in youth political radicalism. In that research Gavray et al. (2012) showed that among Belgian adolescent boys, illegal political behavior was associated with the perception of personal discrimination. Šerek et al. (2018) showed that poor quality of relationships with classmates was not associated with youth’s intentions to participate in non-normative political activities. Another two studies analyzed cases of school shootings perpetrated by adolescents. One of them showed that in nearly all cases of school shootings in the United States that happened between 1994 and 2001 radical action was preceded by incidents of peer harassment at school (Leary et al., 2003). Another qualitative study examined adolescent German school attackers and showed that nearly all attackers felt harassed by their peers (Böckler et al., 2018). These studies inform our research only indirectly as they are not focused specifically on political aggression, and it is unclear to what extent their results generalize to a political domain.

While research on individual harassment and radicalization is scarce, there are reasons to expect that harassment might predispose adolescents for political radicalism by lowering their self-esteem and sense of belonging (Juvonen et al., 2000; Stevens & Thijs, 2018) as well as by increasing their self-uncertainty and lack of meaning in life (Henry et al., 2014). Unfortunately, the effects of peer harassment have not been studied on a larger scale and, thus, its role in the process of youth radicalization remains unclear. Moreover, it is unclear whether only an experience of being a direct victim of harassment matters for radicalization or whether aggressive social norms present in one’s context (e.g., classroom) can also prompt individuals to become more aggressive themselves. This would be in line with studies showing that when social norms among peers are conducive toward aggression the threshold for individual aggression lowers as well (e.g., Webber et al., 2013).

## Protective Factors Against Youth Political Radicalism

Social ecological model suggests that whether or not adolescents engage in certain behaviors due to the risk factors present in one social context depends on the presence of protective factors in other contexts (Bronfenbrenner, 1977). In line with this, positive social aspects of the school context have been shown to counteract the negative effects that peer or parental prejudice has on youth attitudes (McGuire et al., 2015; Miklikowska, 2017; Miklikowska et al., 2019). This model suggests that good relationships with important adult figures in adolescents’ lives, such as teachers or parents, might help prevent radicalization by offsetting the negative psychosocial consequences of peer harassment.

This expectation is in line with significance quest theory suggesting that experiences that boost social belonging would reduce the frustration of *significance needs* caused by peer harassment, while rejection at home or by teachers would acerbate the feelings of significance loss (Kruglanski et al., 2018). From the motivational perspective, the more important the source of significance the greater the effects of rejection (vs. acceptance) should be. Given that development of self-worth is fostered by experiences of relatedness to socializing figures (Deci & Ryan, 1991), knowing and feeling that there are significant figures one can turn to in difficult times is conducive to greater self-worth. Moreover, good relationships with parents or teachers might provide youth with alternative means for channeling their *needs frustration* in socially approved ways instead of turning to radicalism.

Indeed, studies have shown that supportive relationships with parents and teachers have consequences for youth political outcomes. For example, supportive relationships with parents have been linked to higher political activism (Miklikowska, 2016) and supportive relationships with teachers have been linked to greater political attentiveness (Jugert et al., 2018) and more positive attitudes toward immigration (Miklikowska et al., 2019). More importantly, it is by counteracting the loss of significance that follows harassment experiences that parents and teachers can counteract the increase in radicalism. Research has shown that parents and teachers play an important role in satisfying *significance needs*, counteracting externalizing behavior, and in processing harassment experiences. Specifically, supportive relationships with parents have been shown to predict increases in youth self-esteem (Allen et al., 1994) and to mitigate the negative effects of ethnic harassment on minority youth self-esteem (Bowes et al., 2010; Sapouna & Wolke, 2013), while low-quality relationships have been shown to predict higher rates of aggressive behavior (Eichelsheim et al., 2010). In similar vein, supportive relations with teachers have been linked to higher youth self-esteem (Ryan et al., 1994), willingness to seek help in times of crisis (Leuschner et al., 2017; Yablon, 2017) and lower rates of delinquency (Wang et al., 2013). Positive relations with teachers also mitigate the negative effects ethnic harassment has on aggressive among minority youth (Yeung & Leadbeater, 2010) and self-esteem (Suárez-Orozco et al., 2009).

## Present Study

Previous research has important gaps that limit our understanding of how political radicalism forms. First, although adolescence seems to be an important period for radicalization, research on youth is limited to a few cross-sectional studies, and attitudinal measures. Second, although significance quest theory underscores the importance of individual harassment experiences, their long-term effects on radicalization have not been studied. At the same time, longitudinal design offers a unique possibility to delineate changes in radicalism and to test the direction of effects between harassment and radicalism. Longitudinal, multilevel design offers an opportunity to compare the effects of individual experiences and classroom-level frustrations (i.e., classroom harassment atmosphere). It is possible that both the experience of being a direct victim of harassment and the presence of aggressive social norm in one’s context (e.g., classroom) prompts individuals to become more aggressive themselves. Third, it is still not clear what factors can prevent youth political radicalism. Although significance quest theory suggests that supportive relations should play a role, no study has examined this.

The goal of this five-wave longitudinal study is to provide better understanding of youth political radicalism by examining (1) the formation of radicalism across the adolescence (13–17), (2) whether peer harassment would predict individual- and classroom-level changes in youth radicalism, and (3) whether supportive relations with teachers or parents would mitigate the negative effects of harassment.

We hypothesized that radicalism would decrease across adolescence and that in years when participants experienced more harassment than they did on average they also engaged in more radical behaviors than their own average (within-person effect). We also expected that adolescents experiencing higher harassment would show a higher level and a slower decrease in radicalism compared to youth with lower harassment (between-person effect). We explored the role of students’ shared perception of classroom harassment (between-classroom effect). On one hand, it could be expected that higher levels of harassment in classrooms would be related to higher radicalism of students in these classrooms due to priming of aggressive social norms. However, it could also be the case that only direct experience of harassment matters for radicalization as only personal experiences have a potential to affect significance motivation. In addition, we expected that the negative effects of peer harassment would be smaller for adolescents perceiving higher teacher or parent support than for youth with less supportive teachers or parents.

## Method

### Participants

The data were collected annually between 2010 and 2014 in seventh largest city in Sweden which resembles the national average on income, unemployment, and ethnic diversity (Statistiska Centralbyrån, 2016). Ten schools were selected from neighborhoods with different ethnic and social backgrounds and every participating class received payment of 100 EUR. The data were collected during school hours and trained assistants administered questionnaires. Adolescents were informed about the voluntary participation, confidentiality of answers, and the types of questions they would answer. Parents received questionnaires by post and 65% of parents responded. About 3.8% of parents refused their children’s participation. This study uses the data that are a part of a larger study on youth political development, Political Socialization Program at YeS (Youth & Society), focused on areas such as political opinions, efficacy, media attention, and contextual influences. Responsible for the planning, implementation, and financing of the collection of data were Profs. Erik Amnå, Mats Ekström, Margaret Kerr, and Håkan Stattin. The data collection was supported by grants from Riksbankens Jubileumsfond. The study was approved by the Regional Ethical Review Board in Uppsala (Dnr 2010/115). The data that support the findings of this study are available from Prof. Erik Amnå. Restrictions apply to the availability of data, which were used with permission for this study. The study was not preregistered. All methods and analysis code are presented in supplementary materials.

Adolescents that formed the initial sample (*N* = 946; *M_ageT1_* = 13.41; *SD* = 0.53; 50.7% girls) had declared varying economic status (*M* = 3.01, *SD* = 0.80; Range 1–4) and ethnic background. About 71.5% were Swedish majority youth and 27.1% youth with an immigrant background, that is, with at least one parent born outside of the Nordic countries. Around 71.0% of the latter category had at least one parent born outside of Europe. Adolescents were nested in 38 classrooms that were stable between T1 and T3. Three classrooms with less than eight participants (*N* = 12) were excluded to eliminate possible bias for aggregated class and individual-level variables. Individuals (*N* = 31) who changed classrooms one time between T1 and T3 were classified as belonging to the classroom where they spent most of their time, while individuals (*N* = 42) who changed classrooms twice between T1 and T3 were excluded. At T4 adolescents transitioned to new schools and were classified to many and different new classes together with students from outside of this sample. The final sample consisted of *N* = 892 adolescents, 51.1% female, nested in 35 classrooms. The data were organized by age: youth 13 to 14 years old at T1 (*M* = 13.41, *SD* = 0.52) and 17 to 18 at T5 (*M* = 17.32, *SD* = 0 .47).

To test whether the dropout of adolescents from T1 to T5 (*N* = 290) was related to the background and the study variables, logistic regression analyses were performed testing whether attrition (dropout = 0, retention = 1) was predicted by the background (gender, family structure [intact vs. not intact], income) or the T1 study variables. The results showed that dropout was not related to the background or study variables with the exception of political radicalism and teacher support. Although, adolescents who reported lower radicalism or perceived their teachers as more supportive were more likely to stay in the survey than adolescents who reported higher radicalism or perceived their teachers as less supportive, χ^2^ (1, *N* = 822) = 7.81, *p* = .005 and χ^2^ (1, *N* = 831) = 4.43, *p* = .035, respectively, low values of Nagelkerke *R*^2^ (*R*^2^ = .013 and *R*^2^ = .007, respectively) indicated that this would have a small chance of affecting the analyses (Borooah, 2001). Overall, no major differences in the study or background variables emerged between those adolescents who stayed in the study and those who dropped out. Inspection of the data for missing values showed that the average of missing data for all study variables was 17.7%. To account for the missing data, the Full Information Maximum Likelihood (FIML) was used. FIML has been shown to provide more reliable standard errors than mean imputation or listwise or pairwise deletion (Little & Rubin, 2002).

### Measures

#### Political radicalism

At all time-points adolescents reported on their radical political behaviors during the last 12 months by rating five items on a 3-point Likert-type scale (1 = *No*, 2 = *Once or twice*, 3 = *Yes, several times*): “Painted political messages or graffiti on walls” (*M_T1_* = 1.09, *SD* = 0.36), “Taken part in an illegal action/demonstration or occupation” (*M_T1_* = 1.09, *SD* = 0.35), ”Taken part in a political event where property was destroyed” ” (*M_T1_* = 1.12, *SD* = 0.41), “Taken part in a political event where there was a confrontation with political opponents or the police” (*M_T1_* = 1.10, *SD* = 0.38), and “Broken the law on political grounds” (*M_T1_* = 1.14, *SD* = 0.43). The mean of the items was used to construct the scale score. Similar items have been used before to tap political radicalism (e.g., Moskalenko & McCauley, 2009). The internal reliabilities for the items in this study were good .93, .93, .84, .82, and .89 for T1 to T5, respectively. Confirmatory factor analysis showed that the items represented one factor at all timepoints. The factor explained on average 70% variance. All factor loadings were above .74 with the average being .84. Loadings above .70 are considered excellent (Brown, 2006).

#### Peer harassment

At all time-points adolescents reported on their experience of harassment by rating two items on a four-point Likert-type scale (1 = *Absolutely disagree* to 4 = *Absolutely agree*): “There are classmates who harass me” and “There are classmates who bully me.” The mean of the items was used to construct the scale score. Similar items have commonly been used before to tap peer harassment (Juvonen et al., 2000). The internal reliabilities for the items in this study were good .80, .81, .80, .85, and .85 for T1 to T5, respectively. These estimates are similar to those reported in previous studies (Juvonen et al., 2000).

#### Perceived teacher support

At all time-points adolescents reported on their perceived teacher support by rating five items such as “Most of my teachers treat me fairly” (item reversed), on a 4-point Likert-type scale (1 = *Absolutely agree at all* to 4 = *Absolutely disagree*). The mean of items was used to construct the scale score. The internal reliabilities for the items in this study were good .73, .77, .79, .79, .83, for T1 toT5, respectively.

#### Perceived parental rejection

At all time-points, adolescents rated six items asking about perceived parental reactions to adolescents doing something that parents do not like such as “Is silent and cold towards you” on a 3-point Likert-type scale (1 = *Never* to 3 = *Usually*). The internal reliabilities for the items in this study were good .71, .79, .84, .84, .78 and .73, .81, .84, .82, .80, for T1 to T5 mother and father, respectively. Given the high correlations between the perceived maternal and paternal rejection (.75, .73, .71, .70, .51), they were collapsed into one scale of perceived parental rejection.

#### Control variables

*Immigrant background* was coded with 1 for individuals with immigrant background (i.e., with at least one parent born outside of Nordic countries) and 0 for native Swedish. *Gender* was coded 1 for females and 2 for males. *SES*. Parents reported on their household’s monthly income on a 7-point scale (1 = *1–10.000 SEK* to 7 = *above 60.001 SEK; M* = 5.06, *SD* = 1.49) and on their education on a scale ranging from 1 = “Less than 9 years of education” to 5 = “University college/University” (*M* = 4.31, *SD* = 0.87). *Perceived Family Finances.* Adolescents reported on their perceived socio-economic status by rating one item “What are your family finances like?” on a 4-point scale ranging from 1 = “My parents always complain that they don’t have enough money” to 4 = “My parents never complain about being short of money” (*M* = 3.01, *SD* = 0.78).

### Preliminary Analyses

Correlations, means, and standard deviations between study variables are reported in Table 1. Correlations between the study and control variables are presented in supplementary materials. Youth experiences of harassment were positively, although weakly, related to their radicalism measured at the same timepoints. Perceived teacher support was negatively related and parental rejection was positively related to harassment and radicalism measured at the same timepoints. One-way analyses of variance showed that youth with immigrant background did not report higher harassment than Swedish majority youth and that boys did not report higher harassment than girls. In contrast, youth with immigrant background reported higher radicalism than Swedish majority youth at T1 and T2, *F*(1, 815) = 9.59, *p* = .002 and *F*(1, 731) = 13.65, *p* < .001, respectively, and boys reported higher radicalism than girls at T1, T3, T4, and T5, *F*(1, 822) = 19.81, *p* < .001, *F*(1, 707) = 28.81, *p* < .001, *F*(1, 620) = 17.97, *p* < .001, and *F*(1, 606) = 7.50, *p* = .006, respectively.

**Table 1.** Means, Standard Deviations, and Correlations Between the Variables.

Variable	*M*	*SD*	1.	2.	3.	4.	5.	6.	7.	8.	9.	10.	11	12	13	14	15	16	17	18	19	20
1. Political radicalism T1	1.11	0.34	—																			
2. Political radicalism T2	1.08	0.31	.14**	—																		
3. Political radicalism T3	1.04	0.18	.12**	.19**	—																	
4. Political radicalism T4	1.03	0.15	.14**	.14**	.40**	—																
5. Political radicalism T5	1.04	0.18	.10**	.10*	.32**	.51**	—															
6. Harassment T1	1.45	0.68	.11**	.13**	.08*	.10**	.01	—														
7. Harassment T2	1.36	0.61	.03	.08*	.06	.18**	.04	.27**	—													
8. Harassment T3	1.31	0.58	.03	.06	.18**	.06	.03	.23**	.35**	—												
9. Harassment T4	1.26	0.53	.11**	.06	.13**	.24**	.13**	.23**	.18**	.26**	—											
10. Harassment T5	1.19	0.46	.07	.12**	.07	.13**	.07	.21**	.19**	.21**	.36**	—										
11. Teacher support T1	3.01	0.52	−.07*	−.13**	−.06	−.05	−.03	−.25**	−.15**	−.19**	−.11**	−.04	—									
12. Teacher support T2	3.02	0.55	−.04	−.10**	−.08*	−.04*	−.10*	−.15**	−.20**	−.21**	−.14**	−.07	.54**	—								
13. Teacher support T3	3.02	0.55	.03	−.08*	−.09*	−.12**	−.09*	−.09*	−.18**	−.30**	−.18**	−.12**	.41**	.57**	—							
14. Teacher support T4	3.17	0.51	−.07	−.04	−.12**	−.05	−.04	−.14**	−.14**	−.23**	−.26**	−.14**	.33**	.35**	.45**	—						
15. Teacher support T5	3.19	0.53	−.01	−.10*	−.09*	−.06	−.09*	−.08*	−.10*	−.21**	−.18**	−.21**	.27**	.30**	.39**	.50**	—					
16. Parental rejection T1	1.28	0.32	.15**	.10*	.03	.15**	.05	.18**	.14**	.10**	.14**	.12**	−.28**	−.20**	−.16**	−.14**	−.09*	—				
17. Parental rejection T2	1.27	0.35	.11**	.08*	.02	.18**	.07	.19**	.20**	.16**	.15**	.12**	−.24**	−.22**	−.20**	−.16**	−.14**	.49**	—			
18. Parental rejection T3	1.36	0.41	.11**	.08*	.20**	.20**	.12**	.13**	.12**	.19**	.13**	.15**	−.18**	−.21**	−.20**	−.23**	−.18**	.33**	.47**	—		
19. Parental rejection T4	1.31	0.37	.04	.15**	.12**	.17**	.11*	.15**	.18**	.16**	.16**	.12**	−.14**	−.15**	−.17**	−.31**	−.17**	.30**	.36**	.39**	—	
20. Parental rejection T5	1.27	0.31	−.01	.03	.07	.16**	.10*	.07	.12**	.11**	.09*	.07	−.11**	−.14**	−.15**	−.24**	−.19**	.27**	.38**	.38**	.56**	—

* *p* < .05. **p < .01.

### Main Analyses

We used Mplus 8 (Muthén & Muthén, 1998-2018) and multilevel models to test whether peer harassment would predict the within- and between-person changes in youth political radicalism. Within-person effects represent the associations between fluctuations in peer harassment between waves (i.e., deviations from own average score on harassment across five waves) with fluctuations in radicalism between waves (i.e., deviations from own average score on radicalism across five waves). In other words, they indicate whether in years when a participant experienced more harassment than they did on average they were also engaged in more radical behaviors than their own average.

Between-persons effects, by contrast, examine differences between individuals (i.e., how adolescents with different levels of harassment differ in radicalism). The time-invariant predictors were grand-mean centered (i.e., based on the sample average of the means from five timepoints) and time-varying predictors were person-mean centered (i.e., based on the individual average of the means from five timepoints).

In the first step, we assessed average change in three consecutive models. Model 1 included random intercept that split the variance in youth radicalism into within-person (i.e., fluctuations in radical behavior within adolescents over time), between-person (i.e., differences in the level of radicalism between adolescents), and between-classroom levels (i.e., differences in the level of radicalism between classrooms). Model 2 included fixed linear and quadratic slopes that represented the average over-time change of youth radicalism. Model 3 included a random slope representing differences in change that vary between persons or classrooms. In the next step, we added predictors of the random intercepts and slopes, that is, harassment at the between- and within-person levels (Model 4).

We specified moderation models at every level to test whether teacher support or parental rejection would moderate the effects of harassment. These models included paths from harassment and teacher support/parental rejection to youth political radicalism and paths from the interaction term between harassment and teacher support/parental rejection to radicalism. A significant path from the interaction term to radicalism would indicate that teacher support or parental rejection moderated harassment effects. We used deviance test (Δ−2LL) and Akaike Information Criterion (AIC) to evaluate all nested models. Kreft and de Leuw (1998) suggest that the model with lower deviance and AIC is the better-fitting model.

## Results

### How Does Youth Political Radicalism Change Over Time?

**Figure 1.**
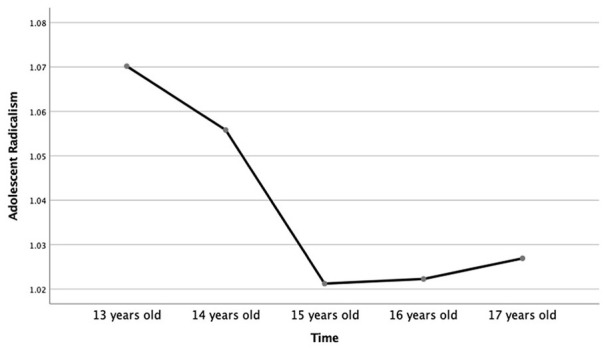
Average developmental change in radicalism across adolescence.

We built a random-intercept model, Model 1, to examine the over-time trend in youth political radicalism. The intraclass-correlations (ICCs) from this model showed that 22% of variance in political radicalism was between adolescents and 0.5% of variance was between classrooms. The remaining variance was on the within-person level. Adding linear and quadratic fixed slopes in Model 2 significantly improved model fit, Δ−2LL = 46.10 (2), *p* = .001; ΔAIC = 42.11. The estimates were *B* = −0.04, *p =* .001, 95% CI [−0.06, −0.03]) and *B* = 0.01, *p =* .004, 95% CI [0.00, 0.01]) for linear and quadratic slope, respectively, showing a small decrease followed by a plateau in radicalism.^1^ The average developmental change in radicalism is illustrated in Figure 1.

Model 3 showed that there was a significant variation around the linear slope at the between-person level (σ² = 0.00, *p =* .001, 95% CI [0.00, 0.00]), indicating that there were differences between youth in their rate of change. There was no significant variation around the linear slope at the classroom level (*B* = 0.00, *p =* .176, 95% CI [0.00, 0.00]) and it was constrained to zero. Setting the linear slope at random at the between-person level improved model fit, Δ−2LL = 185.26 (1), *p* = .001; ΔAIC = 254.84. Table 2 contains all model fit indices and parameter estimates.

**Table 2.** Models to Account for the Change in Adolescents’ Political Radicalism.

Model	Harassment effect models					
	Fit LL (*df*)	Change −2LL (*df*)	L1 Within-person (*B*)	L2 Between-person (*B*)	L3 Between-class (*B*)	Variance
Model 1 Unconditional	−173.26 (4) AIC = 354.53	—	—	—		.053 L1.015 L2.000 L3 Total: .068
Model 2 Fixed linear and quadratic slope L1	−150.21 (6) AIC = 312.42	46.1 (2) *p* = .001	*B*_lin_ = −0.043 *p =* .001 *B*_qd_ = 0.007 *p =* .004	—		.053 L1 .015 L2 .000 L3 Total: .068
Model 3 random linear slope L2	−57.58 (7) AIC=129.17	185.26 (1) *p* = .001	*B*_qd_ = −0.003 *p =* .001			.047 L1 .053 L2 .000 L3 Total: .100
Model 4 Predictors at L1, L2, L3	187.21 (11) AIC= −352.42	489.58 (4) *p* = .001	*B*_qd_ = −0.003 *p =* .002 *B* = 0.022 *p =* .013	*B*_Level_ = 0.14 *p* = .001 *B*_Slope_ = −0.025 *p* = .003	*B*_Level_ = −0.12 *p* = .774	.043 L1 .035 L2 .000 L3 Total: .078

*Note.* AIC = Akaike information criterion. LL = Log-likelihood ratio.

### Does Harassment Predict Within- and Between-Person Changes in Youth Political Radicalism?

In the next step, Model 4, we entered harassment as a predictor of: the average change at the within-person level, the random intercept and slope at the between-person level, and the random intercept at the between-classroom level. This improved model fit, Δ−2LL = 489.58 (4), *p* = .001; ΔAIC = 223.25, compared to the model without any predictors.

At the within-person level, fluctuations in harassment were positively related to fluctuations in youth radicalism (*B* = 0.02, *p* = .013, 95% CI [0.01, 0.04]). That is, in years when adolescents reported higher than average harassment, they reported higher than average radicalism. A closer examination of the direction and timing of effects with RICLPM (Hamaker et al., 2015) showed no significant lagged effects, indicating that within-person changes in radicalism were not predicted by deviations from youth own expected scores on harassment assessed 1 year earlier (or vice versa). However, results showed significant correlated changes between harassment and radicalism, indicating that within-person increases in harassment were linked to instantaneous increases in radicalism. The analyses and results are reported as supplementary material.

**Figure 2.**
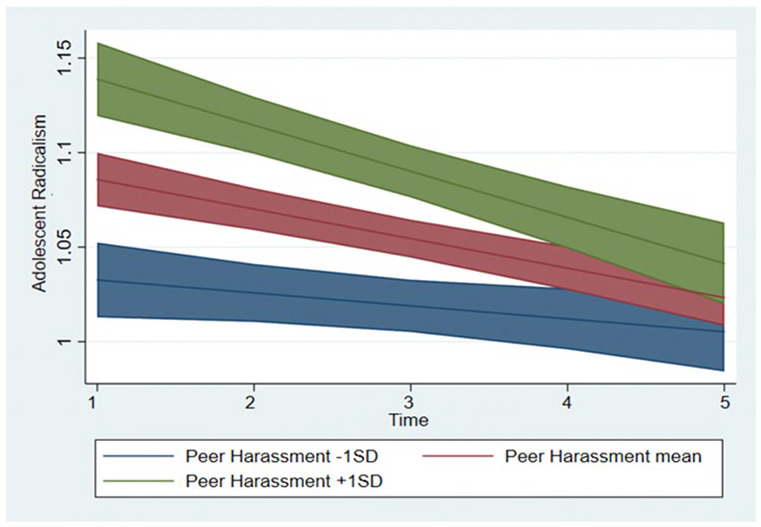
The effects of harassment on the level and change of youth political radicalism. *Note.* Predicted values with 95% confidence intervals.

At the between-person level, harassment significantly predicted a higher level (*B* = 0.14, *p* = .001, 95% CI [0.105, 0.18]) and a more pronounced decrease in radicalism (*B* = −0.02, *p* = .003, 95% CI [−0.04, −0.01]). We transformed the results into standardized betas (Hox et al., 2010). For one standard deviation increase in harassment, the level of youth political radicalism would increase with *β* = .23 and the slope would change by *β* = −.04 standard deviation, respectively, indicating moderate and small effects of harassment, respectively. The effects of harassment on the level and change of youth political radicalism are illustrated in Figure 2.

The effects of harassment on the level and slope of youth political radicalism have not changed when controlling for: parental education (*B* = 0.12, *p* = .001 and *B* = −0.02, *p* = .014), household’s income (*B* = 0.13, *p* = .001 and *B* = −0.02, *p* = .004), and youth perceived socio-economic status (*B* = 0.13, *p* = .001 and *B* = -0.02, *p* = .038). We also tested whether ethnicity (0 = *native Swedish*, 1 = *immigrant background*) and gender (1 = *girl*, 2 = *boy*) would moderate harassment effects. The effects of harassment on the radicalism of immigrant youth were not more pronounced compared to native youth. In contrast, the effects of harassment were stronger for boys than girls. The moderation tests and their results are reported as supplementary material. At the between-classroom level, harassment did not significantly predict the level of radicalism (*B* = −0.12, *p* = .774, 95% CI [−0.77, 0.54]).

In sum, within-person increases in peer harassment were associated with increases in youth radicalism and adolescents reporting higher harassment compared to their peers reported higher political radicalism. There were no significant effects at the classroom level.

### Does Perceived Teacher Support Moderate the Effects of Harassment?

At the within-person level, a model with freely estimated paths from the interaction term between harassment and teacher support and from teacher support to political radicalism was not significantly different from a model where the path from the interaction term was constrained to 0, Δ−2LL = 3.05 (1), *p* = n.s.; ΔAIC = −1.04. The path from the interaction term to political radicalism was not significant (B = −0.04, *p* = .081, 95% CI [−0.07, −0.00]). This means that teacher support did not moderate the effects of peer harassment on radicalism at the within-person level. That is, in years when adolescents reported higher than average teacher support, they did not experience weaker effects of harassment on radicalism. In the model without the interaction, the main effect of teacher support was not significant (*B* = 0.00, *p* = .965, 95% CI [−0.01, 0.01]).

**Figure 3.**
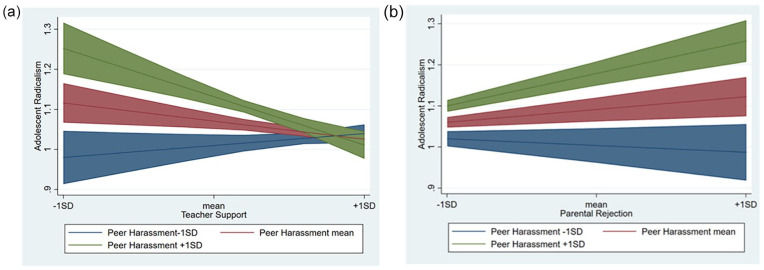
Moderation of harassment effects by teacher support (a) and by parental rejection (b). *Note.* Predicted values with 95% confidence intervals.

At the between-person level, a model with freely estimated paths from the interaction term between harassment and teacher support and from teacher support to the level and slope of political radicalism had a significantly better fit than a model where these paths were constrained to 0, Δ−2LL = 26.62 (2), *p* = .001; ΔAIC = 22.62. The paths from the interaction term to the level and slope of political radicalism were significant (*B* = -0.30, *p* = .001, 95% CI [−0.39, −0.20] and *B* = 0.08, *p* = .001, 95% CI [0.04, 0.11], respectively). This means that teacher support moderated the effects of peer harassment on radicalism at the between-person level. That is, the effects of harassment on the level and change in youth political radicalism were less pronounced for adolescents who, on average, perceived their teachers as more supportive, which is illustrated by Figure 3 (a). In the model without the interaction, the main effects of teacher support were not significant either (*B* = −.021, *p* = .392., 95% CI [−0.06, 0.01] and *B* = −0.04, *p* = .639, 95% CI [–0.01, 0.01] for the level and slope, respectively).

At the between-classroom level, a model with freely estimated paths from the interaction term between harassment and teacher support and from support to the level of political radicalism was not significantly different from a model where these paths were constrained to 0, Δ−2LL = 0.04 (1), *p* = n.s.; ΔAIC = −1.99. The path from the interaction term to the level of political radicalism was not significant (*B* = −0.01, *p* = .946, 95% CI [−0.35, 0.32]). This means that teacher support did not moderate the effects of peer harassment on radicalism at the classroom level. In the model without the interaction, the main effect of teacher support was not significant either (*B* = 0.02, *p* = .536, 95%CI [−0.47, 0.10]).

### Does Perceived Parental Rejection Moderate the Effects of Harassment?

At the within-person level, a model with freely estimated paths from the interaction term between harassment and parental rejection and from parental rejection to youth radicalism fitted data significantly better than a model where the path from the interaction term was constrained to 0, Δ−2LL = 4.45 (1), *p* = .034; ΔAIC = 2.44. The path from the interaction term to radicalism was significant (*B* = 0.04, *p* = .035, 95% CI [0.01, 0.08]). This means that parental rejection exacerbated the effects of peer harassment on radicalism at the within-person level. That is, in years when adolescents reported higher than average parental rejection, they experienced stronger effects of harassment on radicalism. In the model without the interaction, the main effect of parental rejection on radicalism was not significant (*B* = 0.028, *p* = .075, 95% CI [−0.54, 0.02]).

At the between-person level, a model with freely estimated paths from the interaction term between harassment and parental rejection and from parental rejection to the level and slope of radicalism showed an improved model fit over a model where these paths were constrained to 0, Δ−2LL = 33.97 (2), *p* = .001; ΔAIC = 29.98. The paths from the interaction term to the level and slope of youth radicalism were significant (*B* = 0.40, *p* = .001, 95% CI [0.27, 0.53] and *B* = −0.07, *p* = .010, 95% CI [−0.12, −0.03], respectively). This means that parental rejection exacerbated the effects of peer harassment on radicalism at the between-person level. That is, the effects of harassment on the level and change in youth political radicalism were more pronounced for adolescents who, on average, perceived their parents as more rejecting, which is illustrated by Figure 3 (b). In the model without the interaction, the main effects of parental rejection were significant (*B* = .17, *p* = .001, 95% CI [0.10, 0.24] and *B* = .078, *p* = .001, 95% CI [0.04, 0.11] for the level and slope, respectively). This means that parental rejection also was related to the increase in youth radicalism at this level.

## Discussion

This study used a longitudinal and multilevel approach to examine the role of harassment in the formation of political radicalism across the adolescent years (13–17). While past research suggested that adolescence may be a critical period for radicalization and that individual experiences of peer harassment may play a role in this process, we were unable to identify any study that examined this relationship. The current results revealed important insights into the formation of political radicalism in adolescence, the role of peer harassment in the process of radicalization, and the factors that might counteract radicalization.

### The Mean Level and Change in Youth Political Radicalism Throughout Adolescence

First, it is important to note that the overall level of youth radicalism in our study was low. Across different political behaviors fewer than 10% of the sample engaged in them at all. It is not surprising given that, in the general population, few people engage in extremisms of any kind (Kruglanski et al., 2017). While it does not make this phenomenon less important, given the social consequences of radicalisation, our results should be interpreted in this context.

Second, the results indicated that political radicalism decreased from early to mid-adolescence, and plateaued thereafter. These results are in line with other studies on youth demonstrating fluctuations in radical beliefs over that period and decreases in externalizing behavior. The decrease in radicalism could indicate a maturation process, where adolescents gain more cognitive control over their behavior, a regulation of emotional reactions to negative social stimuli such as social rejection (Casey & Caudle, 2013; Silvers et al., 2012), and the development of a more stable sense of identity (Crone, et al., 2016). This decrease could also reflect a diminishing peer influence in general (Steinberg & Monahan, 2007) and declining associations with delinquent peer groups in particular (Elliott & Menard,1996; Lacourse et al., 2003; Warr, 1993). Alternatively, this decrease could be driven by the educational structure. A transition to high school between mid and late adolescence implies a dissolution of peer ties that facilitate radical activism and introduces a new set of goals and challenges that may compete with political activity. Thus, it would be important to verify this pattern of results in settings without a major school transition.

Higher levels of radical activism among younger adolescents suggest that younger adolescents might be particularly easy targets of radical mobilization because of their already heightened predisposition for deviant action. This however does not mean that younger adolescents prefer radical tactics over other forms of activism. Cross-sectional group comparisons show that hard tactics are less attractive to the young than milder forms of unconventional political behavior, such as civil disobedience (Watts, 1999). It is worth stressing that even in our sample, the overall level of engagement in radical actions was low, which is typical for nonextremist samples.

### The Role of Peers’ Harassment

Third, the results showed that youth political radicalism was related to peer harassment at the within- and between-person level. On the within-person level, fluctuations in harassment between waves (i.e., deviations from own average score on harassment across all waves) were positively related to fluctuations in youth political radicalism (i.e., deviations from own average score on radicalism across all waves). That is, in years when adolescents experienced more harassment than they usually do, they contemporaneously increased in political radicalism. The positive link between higher harassment and radicalism was also found on the between-person level, where peer harassment significantly predicted differences in radicalism between adolescents. Specifically, youth who on average (i.e., across all waves) experienced more peer harassment compared to their peers showed higher average levels of and more pronounced decrease in radicalism compared to their peers who experienced less harassment. This result illustrates a slow formation of different outcomes for different adolescents (i.e., experiencing lower or higher levels of harassment). Although a finding that students with more peer harassment showed a steeper decline in radicalism could seem inconsistent with their overall greater political radicalization, it is most likely due to the fact that those who experienced low harassment at the first wave had a low starting level of political radicalism. Since their radical political behavior was already very low they could not show as strong a decrease as youth who experienced high harassment.

Overall, these results are in line with significance quest theory suggesting that political radicalism is a result of a low sense of personal significance, which is a consequence of harassment experiences (Kruglanski et al., 2013, 2018). Being a victim of peer harassment is a source of insignificance, and it may facilitate interest in radical behaviors as means of mitigating these feelings. These findings are also in line with research showing significant associations between peer harassment and aggressive radicalization (Böckler et al., 2018; Leary et al., 2003; Lyons-Padilla et al., 2015). They extend this research by showing that youth experiences of peer harassment are associated with instantaneous (rather than lagged) changes in their political radicalism and that highly harassed youth have higher radicalism across adolescent years compared to their less-harassed peers. They also show that the effects of harassment were small-to-moderate. Together with previous studies, these results offer support to claims that harassed youth are more likely to engage in political radicalism.

At the same time, the atmosphere of harassment in the classroom did not seem to play a role. Youth from classes where students perceived more harassment were not significantly different in their radicalism than youth from classrooms with less harassment. This result contributes to the existing literature on radicalism by suggesting that it is a personal experience of harassment (i.e., at the within- and between-person level) that matters for political radicalism rather than being embedded in a context where harassment is prevalent (but not experienced personally). This suggests that when harassment is an idiosyncratic experience, but not only a norm within an ingroup, it is more likely to translate into radicalization. It is in line with the motivational account, according to which personal needs and their frustration play a central role and social norms act as a moderator when relevant motivation is activated.

In addition, the results showed that effects of harassment were not more pronounced for ethnic minority youth. Although previous studies have shown links between ethnic harassment and attitudinal support for political radicalism (Doosje et al., 2013; Ellis et al., 2015; Lyons-Padilla et al., 2015), our results show that individual harassment is also related to radicalism. Given that most of the existing research focuses on group discrimination as a motive for radicalism, more studies on the political consequences of individual harassment are needed. Although ethnicity the effects of harassment did not differ depending on youth ethnicity, they were more pronounced for boys than girls. This might be related to different processing of harassment among males and females. It might generate mental and emotional strain that females, due to gender-role stereotyped socialization, respond to with internalizing whereas males respond with externalizing behaviors (Jang, 2007). In line with this, Gavray et al. (2012) showed that females had lower level of illegal political activism than males and that their illegal activism was not linked to personal discrimination, which was the case for males. Finally, the data revealed a decline in peer harassment across adolescence. This is in line with studies showing that, globally, the proportion of students who report being bullied declines with increasing age (United Nations Educational, Scientific and Cultural Organization, 2019).

### The Mitigating Effects of Supportive Relationships With Teachers and Parents

Fourth, this study showed that the effects of peer harassment were moderated by parental rejection at the within- and between-person level. That is, in years when parents were less rejecting, the effects of harassment on youth political radicalism were lower and adolescents who experienced their parents as less rejecting were less affected by peer harassment compared to youth with more rejecting parents. These results are in consonance with research showing that need-supportive parenting fosters youth self-esteem (Allen et al., 1994) and mitigates the negative effects of ethnic harassment on self-esteem (Bowes et al., 2010; Sapouna & Wolke, 2013). Together with these studies, the current results suggest that rejecting parents, by additionally frustrating *significance needs*, might exacerbate the effects of peer harassment.

The effects of peer harassment were also moderated by teacher support at the between-person level. That is, adolescents who experienced their teachers as more supportive were less affected by peer harassment compared to youth with less-supportive teachers. These mitigating effects of support are in consonance with previous research suggesting that teacher support fosters youth self-esteem (Ryan et al., 1994) and buffers against peer harassment by restoring sense of self-worth (Suárez-Orozco et al., 2009). They are also in line with studies suggesting that teachers can help adolescents make positive adjustments in dealing with harassment and crisis (Leuschner et al., 2017; Yablon, 2017). The absence of teacher effect at the within-person level could indicate that longer exposure to teacher support is needed for it to counteract the effects of peer harassment. Unlike parents, who are a crucial social context with more immediate effects, teachers are a more distal context whose effects might require more time and be more stable. In addition, our measure of teacher support captured an average perception of various teachers at school, which might also explain why fluctuations over time were less relevant in this case.

Taken together, the current results suggest that adolescents experiencing supportive relations may be less affected by peer harassment, which underscores the importance of active strategies aimed at creating good parent–child and teacher–student relationships in preventing radicalization. To offset the negative consequences of harassment, parents and teachers could be encouraged to make youth feel that they are responsive to their problems, and care about their well-being. These results are in line with significance quest theory. Given that parents and teachers are important contacts for youth, their acceptance and support can be an important source of significance, parents more so than teachers.

Importantly though, the main effects of teachers on youth radicalism were not significant, and the main effects of parents were present at the between- but not within-person level. Although from the perspective of significance quest theory, we would expect to obtain those effects as well, these findings may suggest a more nuanced picture of sources of significance. First, it could indicate that the motivational impact of social rejection/support is different depending on how important the social context is for an individual. Greater relative roles of peer and parent contexts followed by the teacher context would be in line with such an assumption. Second, different effects for teachers depending on the level of analysis could suggest that it is a longer exposure to the perceived quality of a relationship that is more important than small fluctuations in a rather stable relationship quality. However, both interpretations are post hoc and would therefore require future investigations.

### Strengths and Limitations

This study has various strengths, such as drawing attention to the role of individual psychological experiences such as peer harassment (rather than purely societal factors) in radicalization, longitudinal assessment, and differentiation of within- and between-person processes as well as classroom-level experiences. Moreover, this study is unique in that it focuses on adolescence, a sensitive but understudied period for radicalization and that it pays attention to the role of peers and protective factors mitigating the effects of peer harassment (parents and teachers). These largely nonselected social contexts exert most influence in adolescent years.

Although longitudinal design enabled us to analyze the effects of harassment over-time and in real-life settings, we cannot make causal inferences. Although experiments testing the immediate effects of a few harassment strategies are plausible (Reijntjes et al., 2011), it is impossible to set up an experiment capturing long-term consequences of harassment. Still, studies could complement our results with games simulating harassment or peer rejection among adolescents (e.g., Bäck et al., 2018). Due to the lack of lagged effects, we cannot speak firmly about the direction of effects. However, correlated over-time changes between those two variables show that increases in harassment are accompanied by instantaneous increases in radicalism. Perhaps with shorter lags between measurement points, for example of 6 months instead of 1 year, we could capture these lagged effects as well. Although we found that radicalism decreases across adolescence, these findings should be seen as exploratory, given the dearth of research on age-related trends.

Data were self-reported and third variables could explain the relationships at the between-person level. It could be argued that adolescents who suffer from depression may exaggerate victimization experiences (De Los Reyes & Prinstein, 2004), receive less support, and be more motivated to engage in radicalism. Unfortunately, we have no indicators of youth depression in these data. Yet, we have controlled for key variables related to youth background, including SES, gender, and ethnicity and results were unchanged. Thus, these processes are unlikely to undermine our results. Although research shows no differences in prevalence of mental illness in terrorists compared to noncriminal groups (Gøtzsche-Astrup & Lindekilde, 2019), future research should examine how milder mental-health issues might interact with harassment experiences in the process of radicalization.

Given the lack of direct measures of personal significance needs, we were not able to test the mechanisms underlying the effects of harassment. Future research would do good to examine the extent to which *significance loss* helps to explain these effects. In addition, the harassment scale captured only self-reported perceptions. Previous research has shown moderate correlations between self- and peer-reported victimization, possibly due to self-under-reporting because of stigma or fear of repercussions (Bouman et al., 2012). Using peer reports might reveal even stronger effects of harassment.

Although the results suggest that radicalization decreases in late adolescence, research on antisocial behavior shows variation around average changes depending on the concrete behavior studied, population, gender, and social experiences (Broidy, Tremblay, et al., 2003; Wiesner & Windle, 2004). Thus, future research should extend our results by examining various developmental trajectories in radicalization. Such research could also compare the developmental pattern of radicalism with other forms of violent behaviors to distinguish between politically motivated behavior and other types of radical and nonnormative actions (e.g., criminal actions).

It is important to highlight that the behaviors under investigation in our study, while nonnormative and destructive, were not examples of extreme violence. Therefore, the question remains whether the relationship between frustrated needs (e.g., due to social ostracism and harassment) and radicalization is linear or whether it takes different shapes at different levels of extremism.

Finally, the results showed that immigrant youth do not report higher levels of harassment than native youth, which is in line with most studies that show no differences in victimization between natives and immigrants (Fandrem et al., 2009; Monks et al., 2008). Still, studies on racist victimization have found that minority youth scored higher (Jasinskaja-Lahti & Liebkind, 2001; Verkuyten & Thijs, 2002) and future research should complement our results by examining the consequences of racist victimization.

### Conclusions and Implications

This study provides a unique insight into the role of peer harassment in the formation of youth political radicalism. It shows that personal experiences of harassment predicted youth political radicalism but the classroom atmosphere of harassment did not. This study also shows that adolescents who perceived their teachers or parents as supportive were less affected by harassment than youth with less-supportive teachers or parents. These findings offer preliminary support for antiharassment programs at schools as a promising measure to counteract youth radicalization. They also suggest that teachers and parents can offset the negative effects of peer harassment by being supportive of adolescents.

## Supplemental Material

sj-docx-1-psp-10.1177_01461672211070420 – Supplemental material for The Making of a Radical: The Role of Peer Harassment in Youth Political Radicalism Click here for additional data file. Supplemental material, sj-docx-1-psp-10.1177_01461672211070420 for The Making of a Radical: The Role of Peer Harassment in Youth Political Radicalism by Marta Miklikowska, Katarzyna Jasko and Ales Kudrnac in Personality and Social Psychology Bulletin

## Notes

1. To ensure that this trend was not driven by the drop out of adolescents from T1 to T5, we run RMANOVA only with participants who had measurements at all five timepoints. The results remained the same: youth radicalism decreased between T1 and T3 *F*(1, 430) = 13.11, η_p_^2^ = .030, *p* < .001 and plateaued thereafter.
