# Supplementary material for: The Making of a Radical: The Role of Peer Harassment in Youth Political Radicalism
Source: Pers Soc Psychol Bull. 2022 Jan 29;49(3):477–92. doi: 10.1177/01461672211070420 (PMC9903247; doi:10.1177/01461672211070420)
Supplement: sj-docx-1-psp-10.1177_01461672211070420 – Supplemental material for The Making of a Radical: The Role of Peer Harassment in Youth Political Radicalism [file sj-docx-1-psp-10.1177_01461672211070420.docx]

**Supplementary material.**

**Table. Correlations between the Study and Control Variables at T1.**

|  | Political Radicalism | Harassment | Teacher Support | Parental Rejection | Gender | Maternal Education | Parental Income | Perceived Family Finances |
| --- | --- | --- | --- | --- | --- | --- | --- | --- |
| Harassment | .11^**^ | - |  |  |  |  |  |  |
| Teacher Support | -.07^*^ | -.24^**^ | - |  |  |  |  |  |
| Parental Rejection | .14^**^ | .17^**^ | -.27^**^ | - |  |  |  |  |
| Gender | .15^**^ | -.03 | .08^*^ | .01 | - |  |  |  |
| Maternal Education | -.16^**^ | -.07 | .01 | -.04 | .01 | - |  |  |
| Parental Income | -.17^**^ | -.07 | .05 | -.07 | .02 | .43^**^ | - |  |
| Perceived Family Finances | .04 | -.09^**^ | .07^*^ | -.11^**^ | .10^**^ | .11^**^ | .23^**^ | - |
| Immigrant Background | .10^**^ | .01 | -0.04 | .10^**^ | -0.03 | -.25^**^ | -.31^**^ | .03 |

** p < .01, * p < .05

**Direction of effects at the within-person level (RI-CLPM).**

To test the possibility of lagged effects and the direction of these effects at the within-person level, Random-Intercept Cross-Lagged Panel Model was estimated (Hamaker, et al., 2015). Following the procedures described by Hamaker and colleagues, each observed score was regressed on its own latent factor (each loading constrained at 1). The stability paths, cross-lagged effects, correlated residuals at T2-T5, as well as within-time correlations at T1 were specified between these latent constructs. Two overarching random intercept factors were added to capture the trait-like differences between persons in harassment and radicalism. The observed scores were the indicators of these factors, and all factor loadings were constrained at 1. The measurement error variances of the observed scores were constrained to zero. Thus, all variation in the observed measures was completely captured by within-person and between-person latent factor structure. Figure features all the paths of the model.

The fit of the initial, the model was good, χ^2^(23) = 33622, *p* = .070, RMSEA = .023, SRMR = .028. At the between-person level, there was a strong correlation between peer harassment and radicalism r = .50, *p* = .001). This indicates that adolescents who reported higher levels of harassment across five time points reported more radicalism across five years. After controlling for these stable, trait-like differences, some evidence was found for small within-person associations. There were significant correlated residuals between peer harassment and radicalism at T1, T2, and T3 (r = .035, *p* = .038; r = .044, *p* = .036; r = .096, *p* = .036, respectively), indicating that a within-person changes in peer harassment were linked to within-person changes in radicalism. No significant lagged effects were found.

**
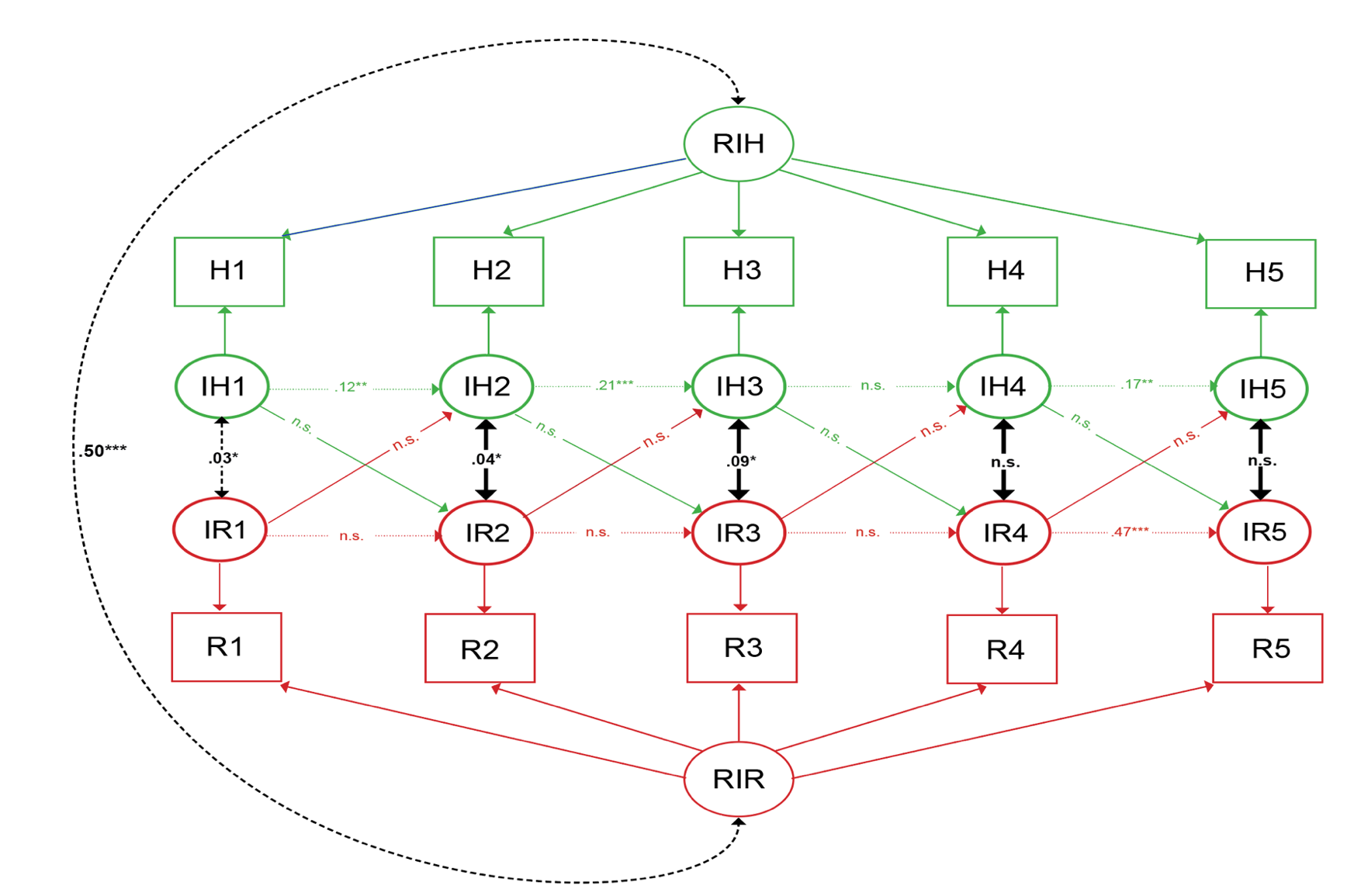
Figure. Random Intercept Cross-Lagged Panel Model**. Latent constructs (σ^2^ within person): IH = harassment; IR = radicalism. Observed scores: H = harassment; R = radicalism. Random intercepts (σ^2^ between-person): RIH = harassment; RIR = radicalism. Solid line = lagged paths; Bold line = correlated residuals; Dashed line = correlations; Dotted line = stability paths.

**Moderation of harassment effects by ethnicity and gender.**

To test whether ethnicity (0 = native Swedish, 1 = immigrant background) would moderate the effects of harassment, moderation model was specified at the between-person level. A model with freely estimated paths from the interaction term between harassment and ethnicity to the level and slope of youth political radicalism and from ethnicity to the level and slope of youth radicalism was not significantly different from a model where the paths from the interaction term to the level and slope were constrained to zero (Δ-2LL = 5.48 (2), *p* = n.s.; ΔAIC = 1.48), indicating that the effects of harassment were not more pronounced for immigrant youth. The main effect of ethnicity was significant for the level of youth radicalism (B = 0.09, *p* = 001, 95%CI [0.05, 0.12]) and for the slope (B = -0.03, *p* = .001, 95%CI [-0.04, -0.02]), indicating that immigrant youth reported higher level and bigger decrease in radicalism compared to natives.

To test whether gender (1 = girl, 2 = boy) would moderate the effects of harassment, moderation model was specified at the between-person level. A model with freely estimated paths from the interaction term between harassment and gender to the level and slope of youth radicalism and from gender to the level and slope of youth radicalism showed a significantly improved model fit over a model where the paths from the interaction term to the level and slope were constrained to zero (Δ-2LL = 20.18 (2), *p* = .001; ΔAIC = 16.18). The path from the interaction term between harassment and gender to the level of youth radicalism was significant (B = 0.125, *p* = .007, 95%CI [0.05, 0.20], respectively). The path from the interaction term between harassment and gender to the slope of youth radicalism was not significant (B = - 0.01, *p* = n.s., 95%CI [-0.035, 0.02]. The main effects of gender were significant for the level (B = 0.08, *p* = .001, 95%CI [0.06, 0.10]) and slope (B = - 0.02, *p* = .001, 95%CI [-0.02, -0.01), indicating that boys reported higher level and bigger decrease in radicalism that girls. An inspection of the moderation showed that the effect of harassment on the level of youth radicalism was stronger for boys than girls.

**Methods**

**Political Radicalism.**

*Have you done any of the following during the last 12 months?*

| **Item** | **Response** |
| --- | --- |
| Painted political messages or graffiti on walls? | **1** No  **2** Yes, occasionally  **3** Yes, several times |
| Taken part in an illegal action/demonstration or occupation? | **1** No  **2** Yes, occasionally  **3** Yes, several times |
| Taken part in a political event where property was destroyed? | **1** No  **2** Yes, occasionally  **3** Yes, several times |
| Taken part in a political event where there was a confrontation with political opponents or the police? | **1** No  **2** Yes, occasionally  **3** Yes, several times |
| Broken the law on political grounds? | **1** No  **2** Yes, occasionally  **3** Yes, several times |

**Peer Harassment.**

*I have classmates who…*

| **Item** | **Response** |
| --- | --- |
| There are classmates who harass me. | **1** Absolutely agree  **2** Agree  **3** Don’t agree  **4** Absolutely disagree |
| There are class mates who bully me. | **1** Absolutely agree  **2** Agree  **3** Don’t agree  **4** Absolutely disagree |

**Perceived Teacher Support.**

*I have teachers who…*

| **Item** | **Response** |
| --- | --- |
| Most of my teachers treat me fairly. | **1** Absolutely agree  **2** Agree  **3** Don’t agree  **4** Absolutely disagree |
| Most of my teachers listen to what I have to say. | **1** Absolutely agree  **2** Agree  **3** Don’t agree  **4** Absolutely disagree |
| There are scarcely any teachers who I can talk with if I have problems with something at school. | **1** Absolutely agree  **2** Agree  **3** Don’t agree  **4** Absolutely disagree |
| Most teachers are keen that their students feel good. | **1** Absolutely agree  **2** Agree  **3** Don’t agree  **4** Absolutely disagree |
| There are scarcely any teachers who praise me when I do a good job. | **1** Absolutely agree  **2** Agree  **3** Don’t agree  **4** Absolutely disagree |

**Perceived Parental Rejection.**

*What do your mother and father do if you do something they don’t like?*

| **Item** | **Response** |
| --- | --- |
| Is silent and cold towards you. | **1** Never  **2** Sometimes  **3** Usually |
| Ignores what you have to say if you try to explain. | **1** Never  **2** Sometimes  **3** Usually |
| Doesn’t talk to you for a long while. | **1** Never  **2** Sometimes  **3** Usually |
| Disregards your points of view or ideas. | **1** Never  **2** Sometimes  **3** Usually |
| Makes you feel guilty for a long time. | **1** Never  **2** Sometimes  **3** Usually |
| Avoids you. | **1** Never  **2** Sometimes  **3** Usually |

**Household’s income.**

| **Item** | **Response** |
| --- | --- |
| What is your household’s´ monthly income? | **1** 1-10,000 SEK  **2** 10,001-20,000 SEK  **3** 20,001-30,000 SEK  **4** 30,001-40, 000 SEK  **5** 40,001-50,000 SEK  **6** 50,001-60,000 SEK  **7** 60,001 SEK – |

**Parental education.**

| **Item** | **Response** |
| --- | --- |
| Which of the following is your highest level of education? | **1** Less than 9 years of study  **2** Compulsory school  **3** High school – vocational  **4** High school – academic  **5** University college/University |

**Perceived family finances.**

| What are your family finances like? | **1** My parents always complain that they don’t have enough money  **2** It happens often that my parents complain that they are short of money  **3** My parents seldom complain about being short of money  **4** My parents never complain about being short of money |
| --- | --- |

**Code**

**1. Model 1**

USEVARIABLES IS

RAD_OT;

CLUSTER = classroom13 ourid;

MISSING ARE ALL (999);

ANALYSIS:

TYPE = THREELEVEL RANDOM;

ESTIMATOR IS ML;

MODEL:

%within%

RAD_OT;

%between ourid%

RAD_OT;

%between classroom13%

RAD_OT;

OUTPUT:

SAMPSTAT CINTERVAL STANDARDIZED tech1;

**2. Model 2**

USEVARIABLES IS

RAD_OT wave0 Qwave;

CLUSTER = classroom13 ourid;

MISSING ARE ALL (999);

within = wave0 Qwave;

ANALYSIS:

TYPE = THREELEVEL RANDOM;

ESTIMATOR IS ML;

MODEL:

%within%

RAD_OT;

RAD_OT ON wave0;

RAD_OT ON Qwave;

%between ourid%

RAD_OT;

%between classroom13%

RAD_OT;

OUTPUT:

SAMPSTAT CINTERVAL STANDARDIZED tech1;

**3. Model 3**

USEVARIABLES IS

RAD_OT wave0 Qwave;

CLUSTER = classroom13 ourid;

MISSING ARE ALL (999);

within = wave0 Qwave;

ANALYSIS:

TYPE = THREELEVEL RANDOM;

ESTIMATOR IS ML;

MODEL:

%within%

RAD_OT;

S1 |RAD_OT ON wave0;

RAD_OT ON Qwave;

%between ourid%

RAD_OT;

s1;

s1 WITH RAD_OT;

%between classroom13%

RAD_OT;

s1@0;

[s1@0];

s1 WITH RAD_OT@0;

OUTPUT:

SAMPSTAT CINTERVAL STANDARDIZED tech1;

**4. Model 4**

USEVARIABLES IS

RAD_OT wave0 Qwave HARR_OT gc_HARC_mean gc_HARC_class;

CLUSTER = classroom13 ourid;

MISSING ARE ALL (999);

between = (ourid) gc_HARC_mean;

between = (classroom13) gc_HARC_class ;

within = wave0 Qwave HARR_OT;

ANALYSIS:

TYPE = THREELEVEL RANDOM;

ESTIMATOR IS ML;

MODEL:

%within%

RAD_OT;

RAD_OT ON HARR_OT;

S1 |RAD_OT ON wave0;

RAD_OT ON Qwave;

%between ourid%

RAD_OT;

s1;

s1 WITH RAD_OT;

RAD_OT ON gc_HARC_mean;

s1 ON gc_HARC_mean;

%between classroom13%

RAD_OT;

s1@0;

[s1@0];

s1 WITH RAD_OT@0;

RAD_OT ON gc_HARC_class;

OUTPUT:

SAMPSTAT CINTERVAL STANDARDIZED tech1;

**5. Model controlling for parental education:**

USEVARIABLES IS

RAD_OT wave0 Qwave HARR_OT gc_HARC_mean gc_HARC_class fpa11a;

CLUSTER = classroom13 ourid;

MISSING ARE ALL (999);

between = (ourid) gc_HARC_mean fpa11a;

between = (classroom13) gc_HARC_class;

within = wave0 Qwave HARR_OT;

ANALYSIS:

TYPE = THREELEVEL RANDOM;

ESTIMATOR IS ML;

MODEL:

%within%

RAD_OT;

RAD_OT ON HARR_OT;

S1 |RAD_OT ON wave0;

RAD_OT ON Qwave;

%between ourid%

RAD_OT;

s1;

s1 WITH RAD_OT;

RAD_OT ON gc_HARC_mean fpa11a;

s1 ON gc_HARC_mean fpa11a;

%between classroom13%

RAD_OT;

s1@0;

[s1@0];

s1 WITH RAD_OT@0;

RAD_OT ON gc_HARC_class;

**6. Model controlling for household’s income:**

USEVARIABLES IS

RAD_OT wave0 Qwave HARR_OT gc_HARC_mean gc_HARC_class fpa15;

CLUSTER = classroom13 ourid;

MISSING ARE ALL (999);

between = (ourid) gc_HARC_mean fpa15;

between = (classroom13) gc_HARC_class;

within = wave0 Qwave HARR_OT;

ANALYSIS:

TYPE = THREELEVEL RANDOM;

ESTIMATOR IS ML;

MODEL:

%within%

RAD_OT;

RAD_OT ON HARR_OT;

S1 |RAD_OT ON wave0;

RAD_OT ON Qwave;

%between ourid%

RAD_OT;

s1;

s1 WITH RAD_OT;

RAD_OT ON gc_HARC_mean fpa15;

s1 ON gc_HARC_mean fpa15;

%between classroom13%

RAD_OT;

s1@0;

[s1@0];

s1 WITH RAD_OT@0;

RAD_OT ON gc_HARC_class;

**7. Model controlling for perceived socio-economic status:**

USEVARIABLES IS

RAD_OT wave0 Qwave HARR_OT gc_HARC_mean gc_HARC_class pa8;

CLUSTER = classroom13 ourid;

MISSING ARE ALL (999);

between = (ourid) gc_HARC_mean pa8;

between = (classroom13) gc_HARC_class;

within = wave0 Qwave HARR_OT;

ANALYSIS:

TYPE = THREELEVEL RANDOM;

ESTIMATOR IS ML;

MODEL:

%within%

RAD_OT;

RAD_OT ON HARR_OT;

S1 |RAD_OT ON wave0;

RAD_OT ON Qwave;

%between ourid%

RAD_OT;

s1;

s1 WITH RAD_OT;

RAD_OT ON gc_HARC_mean pa8;

s1 ON gc_HARC_mean pa8;

%between classroom13%

RAD_OT;

s1@0;

[s1@0];

s1 WITH RAD_OT@0;

RAD_OT ON gc_HARC_class;

**8. Moderation by gender:**

USEVARIABLES IS

RAD_OT wave0 Qwave HARR_OT gc_HARC_mean gc_HARC_class kon inter2;

CLUSTER = classroom13 ourid;

MISSING ARE ALL (999);

between = (ourid) gc_HARC_mean kon inter2;

between = (classroom13) gc_HARC_class;

within = wave0 Qwave HARR_OT;

DEFINE:

inter2= kon*gc_HARC_mean;

ANALYSIS:

TYPE = THREELEVEL RANDOM;

ESTIMATOR IS ML;

MODEL:

%within%

RAD_OT;

RAD_OT ON HARR_OT;

S1 |RAD_OT ON wave0;

RAD_OT ON Qwave;

%between ourid%

RAD_OT;

s1;

s1 WITH RAD_OT;

RAD_OT ON gc_HARC_mean;

s1 ON gc_HARC_mean;

RAD_OT ON kon inter2;

s1 ON kon inter2;

%between classroom13%

RAD_OT;

s1@0;

[s1@0];

s1 WITH RAD_OT@0;

RAD_OT ON gc_HARC_class;

OUTPUT:

SAMPSTAT CINTERVAL STANDARDIZED tech1;

**9. Moderation by ethnicity:**

USEVARIABLES IS

RAD_OT wave0 Qwave HARR_OT gc_HARC_mean gc_HARC_class Fpa2abN2 inter2;

CLUSTER = classroom13 ourid;

MISSING ARE ALL (999);

between = (ourid) gc_HARC_mean Fpa2abN2 inter2;

between = (classroom13) gc_HARC_class;

within = wave0 Qwave HARR_OT;

DEFINE:

inter2= Fpa2abN2*gc_HARC_mean;

ANALYSIS:

TYPE = THREELEVEL RANDOM;

ESTIMATOR IS ML;

MODEL:

%within%

RAD_OT;

RAD_OT ON HARR_OT;

S1 |RAD_OT ON wave0;

RAD_OT ON Qwave;

%between ourid%

RAD_OT;

s1;

s1 WITH RAD_OT;

RAD_OT ON gc_HARC_mean;

s1 ON gc_HARC_mean;

RAD_OT ON Fpa2abN2 inter2;

s1 ON Fpa2abN2 inter2;

%between classroom13%

RAD_OT;

s1@0;

[s1@0];

s1 WITH RAD_OT@0;

RAD_OT ON gc_HARC_class;

OUTPUT:

SAMPSTAT CINTERVAL STANDARDIZED tech1;

**10. Moderation by teacher support L1:**

USEVARIABLES IS

RAD_OT wave0 Qwave HARR_OT gc_HARC_mean gc_HARC_class TeacherB_OT inter1;

CLUSTER = classroom13 ourid;

MISSING ARE ALL (999);

between = (ourid) gc_HARC_mean;

between = (classroom13) gc_HARC_class;

within = wave0 Qwave HARR_OT TeacherB_OT inter1;

DEFINE:

inter2 = gc_TEACHERB_mean*gc_HARC_mean;

ANALYSIS:

TYPE = THREELEVEL RANDOM;

ESTIMATOR IS ML;

MODEL:

%within%

RAD_OT;

S1 |RAD_OT ON wave0;

RAD_OT ON Qwave;

RAD_OT ON HARR_OT;

RAD_OT ON TeacherB_OT;

RAD_OT ON inter1;

%between ourid%

RAD_OT;

s1;

s1 WITH RAD_OT;

RAD_OT ON gc_HARC_mean;

s1 ON gc_HARC_mean;

%between classroom13%

RAD_OT;

s1@0;

[s1@0];

s1 WITH RAD_OT@0;

OUTPUT:

SAMPSTAT CINTERVAL STANDARDIZED tech1;

**11.** **Moderation by teacher support L2:**

USEVARIABLES IS

RAD_OT wave0 Qwave HARR_OT gc_HARC_mean gc_HARC_class gc_TEACHERB_mean inter2;

CLUSTER = classroom13 ourid;

MISSING ARE ALL (999);

between = (ourid) gc_HARC_mean gc_TEACHERB_mean inter2;

between = (classroom13) gc_HARC_class;

within = wave0 Qwave HARR_OT;

DEFINE:

inter2 = gc_TEACHERB_mean*gc_HARC_mean;

ANALYSIS:

TYPE = THREELEVEL RANDOM;

ESTIMATOR IS ML;

MODEL:

%within%

RAD_OT;

RAD_OT ON HARR_OT ;

S1 |RAD_OT ON wave0;

RAD_OT ON Qwave;

%between ourid%

RAD_OT;

s1;

s1 WITH RAD_OT;

RAD_OT ON gc_HARC_mean;

s1 ON gc_HARC_mean;

RAD_OT ON gc_TEACHERB_mean;

RAD_OT ON inter2;

s1 ON gc_TEACHERB_mean;

s1 ON inter2;

%between classroom13%

RAD_OT;

s1@0;

[s1@0];

s1 WITH RAD_OT@0;

OUTPUT:

SAMPSTAT CINTERVAL STANDARDIZED tech1;

**12.** **Moderation by teacher support L3:**

USEVARIABLES IS

RAD_OT wave0 Qwave HARR_OT gc_HARC_mean gc_HARC_class gc_TEACHERB_class inter3;

CLUSTER = classroom13 ourid;

MISSING ARE ALL (999);

between = (ourid) gc_HARC_mean;

between = (classroom13) gc_HARC_class gc_TEACHERB_class inter3;

within = wave0 Qwave HARR_OT;

DEFINE:

inter3 = gc_TEACHERB_class*gc_HARC_class;

ANALYSIS:

TYPE = THREELEVEL RANDOM;

ESTIMATOR IS ML;

MODEL:

%within%

RAD_OT;

RAD_OT ON HARR_OT ;

S1 |RAD_OT ON wave0;

RAD_OT ON Qwave;

%between ourid%

RAD_OT;

s1;

s1 WITH RAD_OT;

RAD_OT ON gc_HARC_mean;

s1 ON gc_HARC_mean;

%between classroom13%

RAD_OT;

s1@0;

[s1@0];

s1 WITH RAD_OT@0;

RAD_OT ON gc_HARC_class;

RAD_OT ON gc_TEACHERB_class;

RAD_OT ON inter3;

OUTPUT:

SAMPSTAT CINTERVAL STANDARDIZED tech1;

**13. Moderation by parental rejection L1:**

USEVARIABLES IS

RAD_OT wave0 Qwave HARR_OT gc_HARC_mean gc_HARC_class Parent_OT inter1;

CLUSTER = classroom13 ourid;

MISSING ARE ALL (999);

between = (ourid) gc_HARC_mean;

between = (classroom13) gc_HARC_class;

within = wave0 Qwave HARR_OT Parent_OT inter1;

DEFINE:

inter1 = TEACHERB_OT*HARR_OT;

ANALYSIS:

TYPE = THREELEVEL RANDOM;

ESTIMATOR IS ML;

MODEL:

%within%

RAD_OT;

S1 |RAD_OT ON wave0;

RAD_OT ON Qwave;

RAD_OT ON HARR_OT;

RAD_OT ON Parent_OT;

RAD_OT ON inter1;

%between ourid%

RAD_OT;

s1;

s1 WITH RAD_OT;

RAD_OT ON gc_HARC_mean;

s1 ON gc_HARC_mean;

%between classroom13%

RAD_OT;

s1@0;

[s1@0];

s1 WITH RAD_OT@0;

OUTPUT:

SAMPSTAT CINTERVAL STANDARDIZED tech1;

**14. Moderation by parental rejection L2:**

USEVARIABLES IS

RAD_OT wave0 Qwave HARR_OT gc_HARC_mean gc_HARC_class gc_coldmofa_mean inter1;

CLUSTER = classroom13 ourid;

MISSING ARE ALL (999);

between = (ourid) gc_HARC_mean gc_coldmofa_mean inter1;

between = (classroom13) gc_HARC_class;

within = wave0 Qwave HARR_OT;

DEFINE:

inter1 = gc_coldmofa_mean*gc_HARC_mean;

ANALYSIS:

TYPE = THREELEVEL RANDOM;

ESTIMATOR IS ML;

MODEL:

%within%

RAD_OT;

S1 |RAD_OT ON wave0;

RAD_OT ON Qwave;

RAD_OT ON HARR_OT;

%between ourid%

RAD_OT;

s1;

s1 WITH RAD_OT;

RAD_OT ON gc_HARC_mean gc_coldmofa_mean;

RAD_OT ON inter1;

s1 ON gc_HARC_mean gc_coldmofa_mean;

s1 ON inter1;

%between classroom13%

RAD_OT;

s1@0;

[s1@0];

s1 WITH RAD_OT@0;

OUTPUT:

SAMPSTAT CINTERVAL STANDARDIZED tech1;
